# Phenolic Acids Induce Nod Factor Production in *Lotus japonicus*–*Mesorhizobium* Symbiosis

**DOI:** 10.1264/jsme2.ME21094

**Published:** 2022-03-12

**Authors:** Masayuki Shimamura, Takashi Kumaki, Shun Hashimoto, Kazuhiko Saeki, Shin-ichi Ayabe, Atsushi Higashitani, Tomoyoshi Akashi, Shusei Sato, Toshio Aoki

**Affiliations:** 1 Department of Applied Biological Sciences, Nihon University, Fujisawa, Kanagawa 252–0880, Japan; 2 Graduate School of Life Sciences, Tohoku University, Sendai, Miyagi 980–8577, Japan; 3 Department of Biological Sciences and Kyousei Science Center for Life and Nature, Nara Women’s University, Nara 630–8506, Japan

**Keywords:** *nod* gene inducer, *Lotus japonicus*–*Mesorhizobium* symbiosis, phenolic acids, lipochitooligosaccharides, ultra-high-performance liquid chromatography–tandem-quadrupole mass spectrometry

## Abstract

In legume–rhizobia symbiosis, partner recognition and the initiation of symbiosis processes require the mutual exchange of chemical signals. Chemicals, generally (iso)flavonoids, in the root exudates of the host plant induce the expression of *nod* genes in rhizobia, and, thus, are called *nod* gene inducers. The expression of *nod* genes leads to the production of lipochitooligosaccharides (LCOs) called Nod factors. Natural *nod* gene inducer(s) in *Lotus japonicus*–*Mesorhizobium* symbiosis remain unknown. Therefore, we developed an LCO detection method based on ultra-high-performance liquid chromatography–tandem-quadrupole mass spectrometry (UPLC-TQMS) to identify these inducers and used it herein to screen 40 phenolic compounds and aldonic acids for their ability to induce LCOs in *Mesorhizobium japonicum* MAFF303099. We identified five phenolic acids with LCO-inducing activities, including *p*-coumaric, caffeic, and ferulic acids. The induced LCOs caused root hair deformation, and nodule numbers in *L. japonicus* inoculated with *M. japonicum* were increased by these phenolic acids. The three phenolic acids listed above induced the expression of the *nodA*, *nodB*, and *ttsI* genes in a strain harboring a multicopy plasmid encoding NodD1, but not that encoding NodD2. The presence of *p*-coumaric and ferulic acids in the root exudates of *L. japonicus* was confirmed by UPLC-TQMS, and the induction of *ttsI*::*lacZ* in the strain harboring the *nodD1* plasmid was detected in the rhizosphere of *L. japonicus*. Based on these results, we propose that phenolic acids are a novel type of *nod* gene inducer in *L. japonicus*–*Mesorhizobium* symbiosis.

Leguminous plants are characterized by their ability for symbiosis with a number of Gram-negative bacteria, collectively known as rhizobia. Rhizobia are free-living in soil, but change into bacteroids in the cells of specific host plants, in which they produce ammonium from atmospheric nitrogen and provide it to the host. Host–symbiont recognition and the initiation of symbiosis require the mutual exchange of chemical signals between leguminous plants and rhizobia. In host plants, the processes leading to root nodulation are triggered by rhizobial signal molecules called nod factors (NFs). NFs are lipochitooligosaccharides (LCOs) consisting of the oligomeric backbone of β-1,4-linked N-acetyl-d-glucosamine residues N-acylated at the non-reducing end. Chemical groups such as sulfate, fucose, and acetate, which vary according to the rhizobial strain, may substitute the oligosaccharide backbone (Liang *et al.*, 2014). The transmembrane receptor kinases of the host plant recognize specific NF structures and transmit a signal that triggers a series of symbiotic events, including root hair deformation, the formation and elongation of infection threads, and the induction of nodule primordia (Suzaki *et al.*, 2015). The induction of NF biosynthesis requires specific low-molecular-weight compounds exuded from the roots of the host plant, which are recognized by the rhizobial receptor NodD (Liu and Murray, 2016). When bound to the host-derived ligand, NodD serves as a transcription factor; it binds to *cis* elements called *nod* boxes and induces the transcription of a series of flanking genes, including *nod* genes, which encode enzymes involved in NF biosynthesis (Recourt *et al.*, 1989; Begume *et al.*, 2001; Liu and Murray, 2016). Therefore, these plant factors are called *nod* gene inducers. They include flavonoids and related compounds, such as flavanones, flavones, isoflavones, and chalcones, and function at very low concentrations of 10–100‍ ‍μM (Peters *et al.*, 1986; Kosslak *et al.*, 1987; Hungria *et al.*, 1991). A *nod* box is also located upstream of the rhizobial type III secretion system (T3SS) cluster containing the *ttsI* gene (Okazaki *et al.*, 2010). T3SS secretes effector proteins that affect symbiosis in host plant cells (Okazaki *et al.*, 2013; Sugawara *et al.*, 2018; Kusakabe *et al.*, 2020).

*Lotus japonicus*, together with barrel medic (*Medicago truncatula*) and soybean (*Glycine max*), is a leguminous model system for molecular genetics and genomics (Handberg and Stougaard, 1992; Udvardi *et al.*, 2005; Sato and Tabata, 2006). Data from whole-genome ana­lyses of these species have provided insights into the origin and adaptive evolution of diverse leguminous plants (Cannon *et al.*, 2006; Hougaard *et al.*, 2008; Sato *et al.*, 2008; Young and Bharti, 2012; O’Rourke *et al.*, 2014). A more detailed understanding of symbiotic nitrogen fixation may be obtained by comparing the underlying molecular mechanisms among the three models. The reported *nod* gene inducers that act on *Mesorhizobium* strains, the symbionts of *L. japonicus*, are aldonic acids and related compounds, such as erythronic acid, tetronic acid, and succinic anhydride (Gagnon and Ibrahim, 1998). However, the induction of NF production in *Mesorhizobium* strains requires 10‍ ‍mM tetronic acid or even higher concentrations of erythronic acid and succinic anhydride, more than a thousand-fold higher than those required for NF production in the symbionts of other legumes (Gagnon and Ibrahim, 1998). Furthermore, aldonic acids have not been detected in the root exudates or root tissues of *L. japonicus*. Therefore, the natural *nod* gene inducers of *L. japonicus*–*Mesorhizobium* symbiosis have not yet been identified.

Structural ana­lyses using ultra-high-performance liquid chromatography coupled to quadrupole-time-of-flight (UPLC-QTOF) mass spectrometry (MS) and high-magnetic-field nuclear magnetic resonance spectroscopy revealed that *Mesorhizobium* NFs are a mixture of four major and four minor LCOs that vary in the fatty acid type, the number of carbamoyl groups at the non-reducing end, and the number of acetyl groups attached to fucose at the reducing end (Bek *et al.*, 2010). In the present study, we aimed to identify natural substances that are exuded from *L. japonicus* roots and act on *Mesorhizobium* strains as *nod* gene inducers by directly assaying NF production. We developed an assay method in which LCOs were extracted from small-scale cultures of *Mesorhizobium* strains in the presence of the candidate chemical compound and specifically detected by UPLC–tandem-quadrupole MS (TQMS). UPLC-TQMS is a convenient method for routine assays; however, its resolution is inferior to QTOF MS. We used this method to screen authentic samples of aldonic acids, flavonoids, and related phenolic compounds (instead of the fractionation of plant extracts or root exudates), and then examined the presence of the identified target compounds in the root exudates of *L. japonicus*. We identified five phenolic acids that induce NF production in *Mesorhizobium* strains.

NodD in *Rhizobium leguminosarum* is activated by naringenin, a *nod* gene inducer from *Medicago sativa* (Firmin *et al.*, 1986). When the heterologous *nodD* of *R. leguminosarum* was introduced into *Mesorhizobium japonicum* MAFF303099 (reclassified from *M. loti* based on genome sequence information; Martínez-Hidalgo *et al.*, 2016) via a multicopy plasmid, the application of naringenin induced NF production (López-Lara *et al.*, 1995; Niwa *et al.*, 2001; Bek *et al.*, 2010) and the expression of the *ttsI* gene, the regulator of T3SS (Okazaki *et al.*, 2010). Using a similar approach, namely, the introduction of endogenous *nodD* genes encoded by multicopy plasmids, we herein established which of the two NodD receptors, NodD1 or NodD2, of *M. japonicum* MAFF303099 interacted with the identified phenolic acids.

## Materials and Methods

### Chemicals

The following materials were purchased from the suppliers indicated in parentheses: chlorogenic acid (MP Biomedicals); gossypetin (Indofine Chemical Company); butein, eriodictyol, formononetin, herbacetin, isorhamnetin, kaempferol, myricetin, and quercetin (Extrasynthèse); daidzein and genistein (LC Laboratories); vestitol (Plantech); apigenin, biochanin A, coniferyl alcohol,* o*-coumaric acid, *m*-coumaric acid, coumestrol, 5-hydroxyferulic acid, luteolin, naringenin, phloretic acid, sinapic acid, and umbelliferone (Merck); *p*-coumaric acid, erythronic acid, isoferulic acid, and succinic anhydride (TCI); caffeic acid,* trans*-cinnamic acid, 3,4-dimethoxycinnamic acid, ferulic acid, *p*-methoxycinnamic acid, phenylalanine, tetronic acid, and L-tyrosine (FUJIFILM Wako Pure Chemical). Umbellic acid was prepared from umbelliferone by a treatment with 1 M NaOH at 90°C for 1 h. Isoliquiritigenin was obtained from our laboratory stock (Shimamura *et al.*, 2007).

### NFs (LCOs) and *Mesorhizobium* strains

The plasmid pMP2112 encoding *R. leguminosarum* bv. *trifolii* nodD (López-Lara *et al.*, 1995) and a sample of the NF derived from *M. japonicum* MAFF303099 harboring pMP2112 were provided by H. Kouchi of the International Christian University, Japan. pMP2112 was transferred into *M. japonicum* MAFF303099 (Kaneko *et al.*, 2000; Saeki and Kouchi, 2000).

The bacterial strains and plasmids used in the present study are summarized in Supplementary Table S1. The *nodD1* and *nodD2* deletion (Δ*nodD1-nodD2*) variant of *M. japonicum* MAFF303099 was generated by homologous recombination as described by Hattori *et al.* (2002). The cosmid c243 was digested with *Bam*HI and ligated with a 1.9-kbp *Bam*HI fragment of the kanamycin resistance gene *neo* from pUCKM1 (Saeki *et al.*, 1991). In the resultant knockout plasmid pEMA49, the *nodD1* (*mll6179*)–*nolL* (*mlr8757*)–*nodD2* (*mlr6182*)–*mll6183*–*mlr6185* region was replaced with the *neo* gene. The generated allele was homogenotized with the endogenous genomic locus in *M. japonicum* to produce the Δ*nodD* variant. The construct was verified by Southern hybridization using c243 as a probe.

The *nodA* deletion (Δ*nodA*) variant was constructed essentially as described above, but with the precise in-frame deletion of the NodA coding region. An allele in which *nodA* was replaced with the spectinomycin resistance gene *aadA* was constructed by PCR amplification from pKST001R (Hanyu *et al.*, 2009) with primers to add overhangs (wan_mlr8755_upper and wan_mlr8755_lower). The amplified allele was then exchanged in *Escherichia coli* with the endogenous *mlr8755* allele in the cosmid c242.1 (Hattori *et al.*, 2002) in the presence of the phage lambda Red recombinase (Datsenko and Wanner, 2000) to generate the knockout plasmid pML8755DA. The correct construction of the Δ*nodA* variant was verified by PCR with the primers KS_nodSJ_F01S and KSnodC_Rev01 as well as by Southern hybridization using the wild-type PCR product as a probe.

In the functional ana­lysis of *nodD*, the *nodD1* and *nodD2* genes were introduced separately into *M. japonicum* MAFF303099 and ML033 (Okazaki *et al.*, 2010) as follows. The 1,272-bp fragment containing the coding and promoter regions of *nodD1* (*mlr6182*) and the 1,365-bp fragment containing those of *nodD2* (*mll6179*) were amplified by PCR with the primer pairs pBBR1_nodD1_Fw and pBBR1_nodD1_Rv, and pBBR1_nodD2_Fw and pBBR1_nodD2_Rv, respectively (Supplementary Table S2). PCR products were cloned into pBBR1MCS-2 (Kovach *et al.*, 1995) by In-Fusion HD cloning (Clontech). The plasmids obtained (p*Mj*-NodD1 and p*Mj*-NodD2, respectively) were introduced separately into *E. coli* DH5α and mobilized into *M. japonicum* MAFF303099 using the previously described bacterial conjugation system (Kusakabe *et al.*, 2020). One day after conjugation, transformants containing p*Mj*-NodD1 or p*Mj*-NodD2 were selected on tryptone–yeast-extract plates containing 100‍ ‍μg mL^–1^ phosphomycin and 50‍ ‍μg mL^–1^ kanamycin. Plasmid transfer was confirmed by PCR.

### Culture conditions for *M. japonicum* MAFF303099 and extraction of LCOs

*M. japonicum* MAFF303099 was pre-cultured in TY medium at 28°C overnight. An aliquot of the culture was diluted with fresh TY medium (10‍ ‍mL, OD_660_=0.001) and supplemented with the antibiotics and phenolics shown in Table 1. Regarding *M. japonicum* MAFF303099 carrying pMP2112, antibiotics and naringenin (final concentration, 1‍ ‍μM) were added to the culture medium. Diluted cultures were grown at 28°C for 24 h, centrifuged (8,000×*g*, room temperature, 2‍ ‍min), and LCOs were then extracted from the supernatants with *n*-butanol.

### Extraction of root exudates from legume seedlings

The seeds of alfalfa (*M. sativa*), red clover (*Trifolium pratense*), and *L. japonicus* B-129 Gifu were sterilized with solution containing 2% (v/v) sodium hypochlorite and 0.02% (v/v) Tween-20 for 10‍ ‍min, rinsed five times with sterilized distilled water, and immersed in sterilized distilled water at room temperature overnight. They were then sown in a plastic container containing B&D liquid medium (pH 6.8) and cultivated at 25°C (16 h light/8 h dark) for 7 days. Media containing seedling exudates were collected and loaded onto Oasis HLB cartridges (Waters), and exudate components were eluted with ethanol.

### UPLC-TQMS ana­lysis

The butanol extracts of bacterial cultures and the ethanol extracts of root exudates were concentrated by evaporation, dissolved in 50% acetonitrile, and filtered through polytetrafluoroethylene membrane filters (Merck). UPLC ana­lyses were conducted on Quattro Premier XE (Waters). Separation was performed on an Acquity UPLC BEH C18 column (2.1×100‍ ‍mm, Waters) at 40°C and a flow rate of 0.38‍ ‍mL‍ ‍min^–1^. Gradient elution was performed with 0.1% formic acid in water (A) and 0.1% formic acid in acetonitrile (B) as follows. LCOs: 44% B (0–4‍ ‍min), 44–85% B (4–7‍ ‍min), 85–99.5% B (7–7.1‍ ‍min), 99.5% B (7.1–7.2‍ ‍min); root exudates: 5–15% B (0–7‍ ‍min), 15–99% B (7–10‍ ‍min).

MS spectra were acquired with Quattro Premier v. 4.1 software (Waters) under the following conditions. Qualitative ana­lyses: the selected ion recording (SIR) mode, electrospray ionization (ESI) positive mode, capillary voltage 3.0 kV, cone voltage 30 V, desolvation gas flow rate 800 L h^–1^ at 400°C, cone gas flow rate 50‍ ‍L‍ ‍h^–1^, and source temperature 120°C. Quantitative ana­lyses: the selected reaction monitoring (SRM) mode; conditions described above except that the capillary voltage was 3.5 kV and the cone voltage was 50 V. SRM conditions for phenolic acids in root exudates are shown in Supplementary Table S3.

### Isolation of rhizobia from root nodules of *L. japonicus*

Mature *L. japonicus* plants collected near the coast of Kanagawa, Japan, were dubbed “Bishamon” (34°54'10.3"N 139°53'15.7"E) and “Nojimazaki” (35°08'26.5"N 139°39'36.0"E). Nodules were harvested, and their surfaces were sterilized with solution containing 2% (v/v) sodium hypochlorite and 0.02% (v/v) Tween-20 for 10‍ ‍min and then rinsed five times with sterilized distilled water. Nodules were crushed individually with a pestle in the presence of 40% glycerol, and the homogenates were spread on TY agar medium at 28°C for 10–14 days. A rhizobial colony was isolated from each nodule and named Bishamon1-c2 or Nojimazaki1-a1. Nodule formation by the isolated rhizobia in *L. japonicus* B-129 Gifu was examined according to Aoki *et al.* (2021). The procedure for LCO extraction from rhizobia, namely, Bishamon1-c2, Nojimazaki1-a1, and Tono (Kawaguchi *et al.*, 2002), was the same as that for *M. japonicum* MAFF303099 described above.

### Root hair deformation assay

The root hair deformation assay was performed with the *n*-butanol extract of *M. japonicum* MAFF303099 according to previously described methods, except that B&D liquid and agar media adjusted to pH 6.8 (Broughton and Dilworth, 1971) were used instead of half-strength nitrogen-free HM nutrients (Imaizumi-Anraku *et al.*, 1997). LCOs were extracted with *n*-butanol from cultures of *M. japonicum* MAFF303099 treated with caffeic acid and purified on a solid-phase extraction column. An *n*-butanol extract prepared from mock-treated *M. japonicum* was used as a control. Purified LCOs and the control sample were used to treat 7-day-old *L. japonicus* B-129 Gifu for 24 h, and the roots were stained with toluidine blue and observed by light microscopy.

### Expression ana­lysis of *ttsI*, *nodA*, and *nodB* genes

To analyze the transcriptional regulation of the *ttsI* gene by phenolic acids, we used a chromosomally integrated translational *lacZ* fusion with the ML033 *ttsI* promoter (ML033) as previously described (Okazaki *et al.*, 2010). In the β-galactosidase assay, approximately 50‍ ‍μL of pre-cultured (stationary phase) *Mesorhizobium* strains were inoculated into 5‍ ‍mL of TY liquid medium (OD_660_ 0.01) in 50-mL tubes and grown for 21 h with or without phenolic acids at a final concentration of 10‍ ‍μM.

β-Galactosidase activity was assessed in a microplate assay as previously described (Griffith and Wolf, 2002). The expression of the *nodA* and *nodB* genes was analyzed by qRT-PCR. Pre-cultured (mid-log phase) *Mesorhizobium* strains (1.5‍ ‍mL) were inoculated into 1.5‍ ‍mL of TY liquid medium in 15-mL tubes with or without phenolic acids at a final concentration of 10‍ ‍μM and grown for 4‍ ‍h. Bacterial RNA was stabilized by adding RNAprotect Bacteria Reagent (Qiagen) and extracted with an RNeasy Mini kit (Qiagen), and purified total RNA was then treated with Recombinant DNase I (Takara Bio). cDNA was synthesized from total RNA using an ExScript RT Reagent Kit (Takara Bio). The primer pairs used in the qRT-PCR ana­lysis are listed in Supplementary Table S2. All qRT-PCR measurements were performed in a C1000 Thermal Cycler (Bio-Rad) with a Kapa SYBR Fast qPCR Kit (Kapa Biosystems). The relative expression of the selected genes was calculated as 2^–ΔΔCt^ using the *16S rRNA* gene as a reference. All experiments were performed for three technical and three biological replicates. The primers used to assess the expression levels of the various target genes are listed in Supplementary Table S2.

### Root X-Gal staining assay

*L. japonicus* MG-20 plants were grown for 10 days on 1/2 MS medium agar plates, and ML033, ML033/p*Mj-*nodD1, or ML033/p*Mj-*nodD2 suspensions containing approximately 10^8^ cells were inoculated onto the root surface with low melting point agar containing 0.02% X-Gal. Low melting point agar containing 0.02% X-Gal without rhizobia cells was used as a mock control. Blue staining was observed after a 2-days incubation at 25°C.

## Results

### Screening of compounds for LCO-inducing activity in rhizobia based on direct LCO detection by UPLC-TQMS

**Fig. 1.**
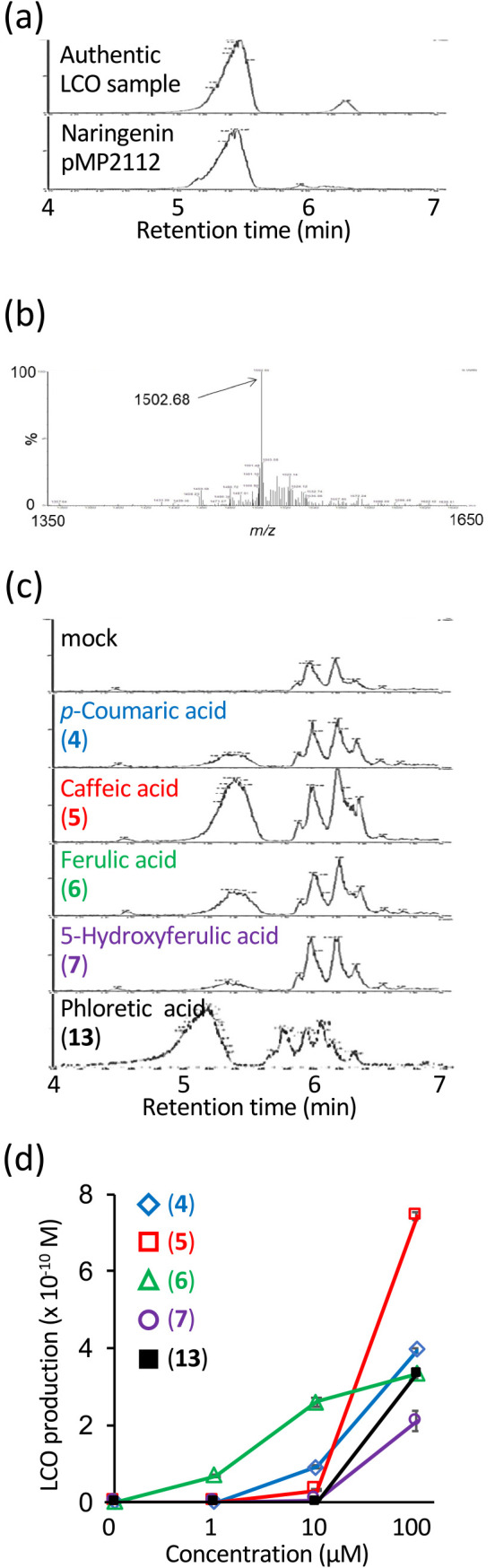
Phenolic acids induce the production of NFs (LCOs) in *Mesorhizobium japonicum* MAFF303099. UPLC-TQMS ana­lysis of an authentic LCO sample and naringenin-induced LCOs in *M. japonicum* harboring pMP2112. Chromatograms recorded by the SIR mode at m/z 1502.7 (a) and the mass spectrum at a retention time of 5.41‍ ‍min (b). (c) UPLC-TQMS ana­lysis of LCOs produced in *M. japonicum* MAFF303099 after the application of the indicated compounds at 15‍ ‍mg L^–1^. (d) LCO production induced by different concentrations of phenolic acids listed in (c). Error bars show S.E. (*n*=4).

To detect NF production with high sensitivity and reproducibility, we established an analytical method to detect LCOs in rhizobial culture media using UPLC-TQMS. When an authentic LCO sample was analyzed in the SIR mode by UPLC-TQMS, a major peak appeared with a retention time of 5–5.5‍ ‍min and a minor peak appeared after 6‍ ‍min (Fig. 1a). To monitor an authentic LCO, we set the *m*/*z* value to 1502.7 based on the consecutive mass spectra of the major peak (Fig. 1b), which we presumed to correspond to previously reported NodMl-V (C18:1, Me, Cb, AcFuc) (López-Lara *et al.*, 1995; Niwa *et al.*, 2001; Bek *et al.*, 2010). The ana­lysis of LCOs extracted from the culture medium of *M. japonicum* MAFF303099 carrying the pMP2112 plasmid, which harbored *nodD* from *R. leguminosarum*, in the presence of 1‍ ‍μM naringenin also revealed a major peak at 5–5.5‍ ‍min (Fig. 1a).

The established analytical method was used to test the LCO-inducing activities of 40 phenolic compounds, including phenylpropanoids, chalcones, flavanones, flavones, flavonols, isoflavones, an isoflavan, and a coumestan, as well as aldonic acids that were previously reported to exhibit *nod* gene-inducing activity (Table 1). LCO induction was analyzed by culturing *M. japonicum* MAFF303099 with 0.3 or 15‍ ‍mg L^–1^ of the tested compounds, followed by *n-*butanol extraction of the culture medium and UPLC-TQMS ana­lyses using the SIR mode at *m*/*z* 1502.7 (Fig. 1b). LCO was produced in the presence of five phenolic acids at 15‍ ‍mg L^–1^: *p*-coumaric acid (**4**), caffeic acid (**5**), ferulic acid (**6**), 5-hydroxyferulic acid (**7**), and phloretic acid (**13**) (Fig. 1c and Table 1). Only ferulic acid (**6**) promoted LCO production at 0.3‍ ‍mg L^–1^ (Table 1). Among the 40 compounds tested, the remaining 35 compounds, including cinnamic acid, produced few or no LCOs (Table 1). We evaluated the reproducibility of LCO-inducing activities at various concentrations of these five phenolic acids employing the SRM mode of UPLC-TQMS and found that their activity increased in different concentration-dependent manners up to 100‍ ‍μM (Fig. 1d). At 1‍ ‍μM, only ferulic acid (**6**) was active, and its activity was the highest among the five inducers at 10‍ ‍μM. In contrast, caffeic acid (**5**) showed weak activity up to 10‍ ‍μM, but was the most active inducer at 100‍ ‍μM. *p*-Coumaric acid (**4**), 5-hydroxyferulic acid (**7**), and phloretic acid (**13**) were only active at 100‍ ‍μM (Fig. 1d).

Using the deletion variants of *M. japonicum* MAFF303099, we confirmed that the LCO-inducing activities of *p*-coumaric acid (**4**), caffeic acid (**5**), and ferulic acid (**6**) depended on the *nodA* and *nodD* genes (Supplementary Fig. S1).

### Phenolic acids induce LCO production in native *Mesorhizobium* strains

**Fig. 2.**
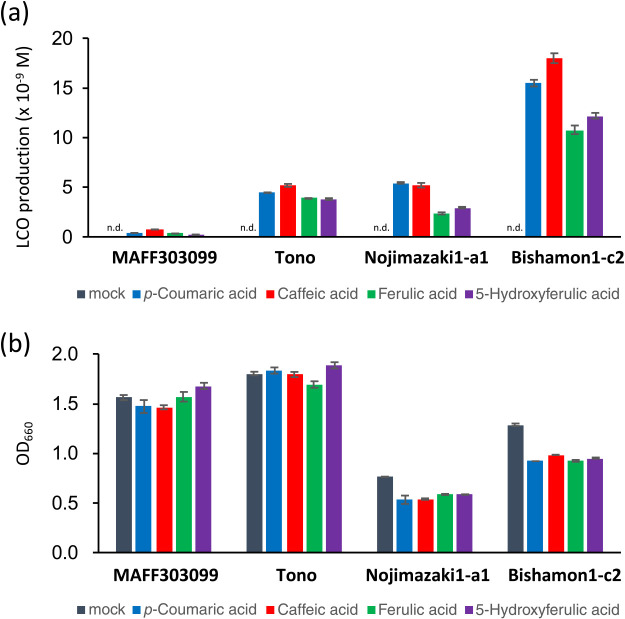
Production of LCOs and growth of *Mesorhizobium* strains isolated from different locations in the presence of phenolic acids. (a) LCO production in *M. japonicum* MAFF303099, Tono, Nojimazaki 1-a1, and Bishamon 1-c2. (b) Growth of the strains tested in panel (a). Error bars show S.E. (*n*=3 or 4).

To investigate whether the phenolic acids identified by screening using *M. japonicum* MAFF303099 generally induce LCOs in native *L. japonicus* rhizobia, we tested three additional *Mesorhizobium* strains isolated from native *L. japonicus*: Tono (Kawaguchi *et al.*, 2002), Nojimazaki 1-a1, and Bishamon 1-c2 (the present study). The phenolic acids tested, namely, *p*-coumaric acid (**4**), caffeic acid (**5**), ferulic acid (**6**), and 5-hydroxyferulic acid (**7**), produced LCOs with good reproducibility and at levels that were significantly higher (25- to 60-fold) in all native *Mesorhizobium* strains than in *M. japonicum* MAFF303099 (Fig. 2a). No adverse effects of these phenolic acids on the growth of rhizobia were observed at the concentrations used (Fig. 2b).

### Biological activities of phenolic acids toward the host plant *L. japonicus*

To examine the abilities of the LCOs produced to function as NFs for *L. japonicus*, we assessed their root hair deformation activities. The root hairs of *L. japonicus* treated with 1 nM LCOs, which were induced by 100‍ ‍μM caffeic acid, showed bending deformation, and some root hair tips were curled (Supplementary Fig. S2a, b, and c). The control sample had no effect on root hairs (Supplementary Fig. S2d).

**Fig. 3.**
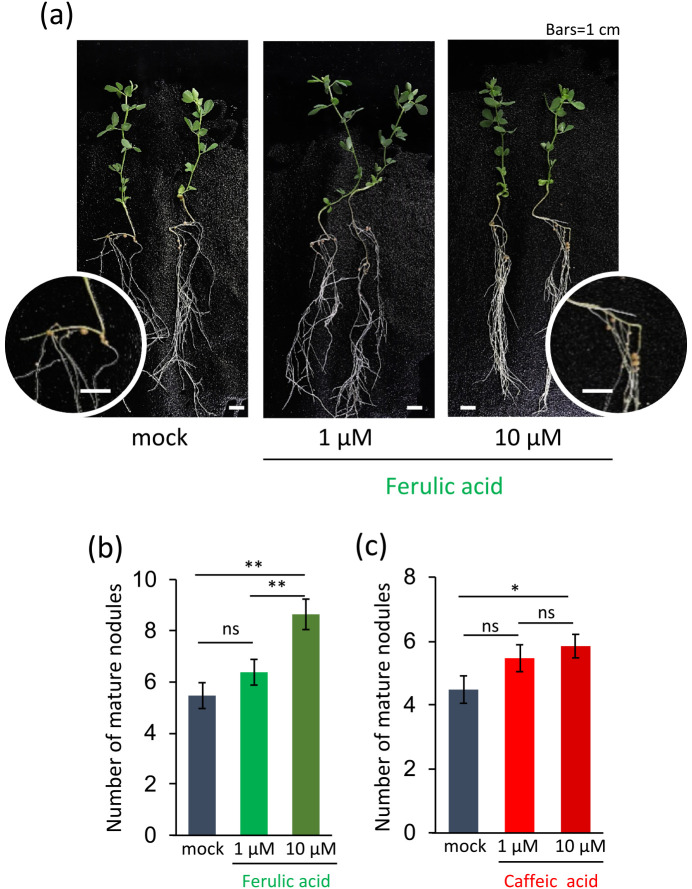
Promotion of nodulation by phenolic acids. (a) Typical images of 4-week-old *Lotus japonicus* MG-20 inoculated with *Mesorhizobium japonicum* MAFF303099 in the absence or presence of ferulic acid. Scale bars: 1‍ ‍cm. (b, c) Number of mature nodules in the absence or presence of (b) ferulic acid or (c) caffeic acid. Error bars show S.E. (*n*=22–27). Significant differences between the absence (mock) and presence of phenolic acids were assessed by the Student’s *t*-test (** *P*<0.01, * *P*<0.05, *ns*, not significant).

Exogenous *nod* gene inducers have been shown to increase the nodule number in pea and soybean (Novák *et al.*, 2002; Pan *et al.*, 2008); therefore, we tested the effects of phenolic acids on *L. japonicus* inoculated with *M. japonicum*. The number of mature nodules significantly increased after 4‍ ‍weeks in the presence of 10‍ ‍μM ferulic acid (Fig. 3a and b) or 10‍ ‍μM caffeic acid (Fig. 3c).

### NodD1 acts as a receptor for phenolic acids

**Fig. 4.**
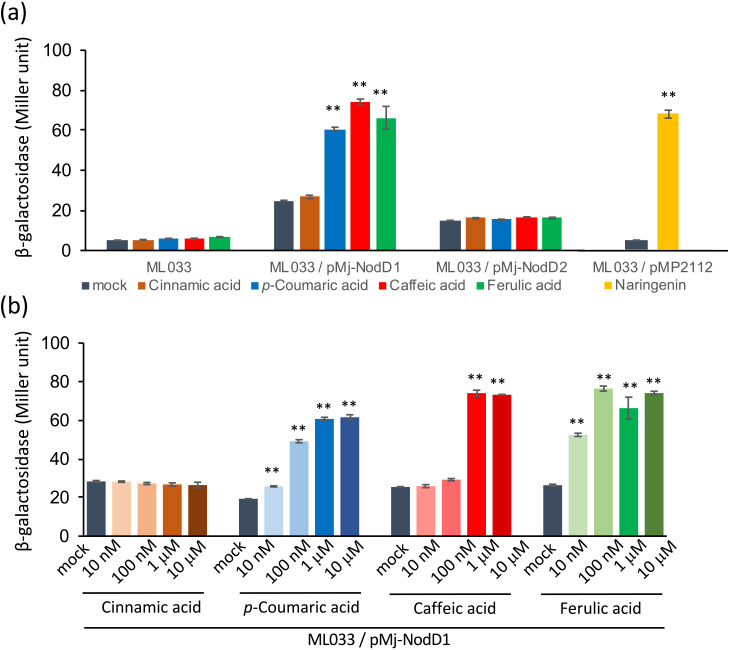
Effects of phenolic acids on the expression of *lacZ*-fused *ttsI*. (a) Effects of harboring p*Mj*-NodD1, the p*Mj*-NodD2 plasmid, or pMP2112 on β-galactosidase induction by the indicated phenolic acids (1‍ ‍μM). (b) β-Galactosidase activity in *Mesorhizobium japonicum* MAFF303099 harboring p*Mj*-NodD1 in the presence of different concentrations of the phenolic acids tested in the panel (a). Error bars show S.E. (*n*=3 or 4). Significant differences between the absence (mock) and presence of phenolic acids were assessed by the Student’s *t*-test (** *P*<0.01).

Rhizobia perceive *nod* gene inducers, such as flavonoids, through their binding to the transcriptional activator NodD, which up-regulates the expression of *nod* genes and the *ttsI* gene, a regulator of T3SS (Firmin *et al.*, 1986; Okazaki *et al.*, 2010). To examine transcriptional responses to the identified phenolic acids, we used *M. japonicum* ML033, in which the translational fusion of *lacZ* with *ttsI* was integrated into the chromosome of *M. japonicum* MAFF303099 (Okazaki *et al.*, 2010). No significant increases in β-galactosidase activity were detected in the presence of 1‍ ‍μM *p*-coumaric acid (**4**), caffeic acid (**5**), or ferulic acid (**6**) (Fig. 4a). We then introduced one of the endogenous *nodD* genes via a multicopy plasmid, followed by an ana­lysis using pMP2112 harboring *nodD* from *R. leguminosarum*. We constructed separate multicopy plasmids harboring *nodD1* (p*Mj-*NodD1) or *nodD2* (p*Mj-*NodD2), and transferred them into *M. japonicum* ML033.

After a 21-h culture with 1‍ ‍μM *p*-coumaric acid (**4**), caffeic acid (**5**), or ferulic acid (**6**), β-galactosidase activity significantly increased in ML033/p*Mj-*NodD1, but not in ML033/p*Mj-*NodD2 (Fig. 4a). Induction levels were similar to that with 1‍ ‍μM naringenin in ML033/pMP2112 (Fig. 4a), which carries *nodD* from *R. leguminosarum* (López-Lara *et al.*, 1995). In contrast, cinnamic acid (**3**), which did not induce LCO production in the screening described above, did not induce β-galactosidase activity in any of the strains (Fig. 4a).

The concentration dependence of β-galactosidase activity in ML033/p*Mj-*NodD1 induced by each phenolic acid (10 nM to 10‍ ‍μM) is shown in Fig. 4b. *p*-Coumaric acid (**4**) and ferulic acid (**6**) both significantly induced activity at the lowest concentration tested (10 nM), whereas caffeic acid (**5**) only induced it at 1 and 100‍ ‍μM. This concentration dependence was similar to that observed in the direct detection of LCO production (Fig. 1).

**Fig. 5.**
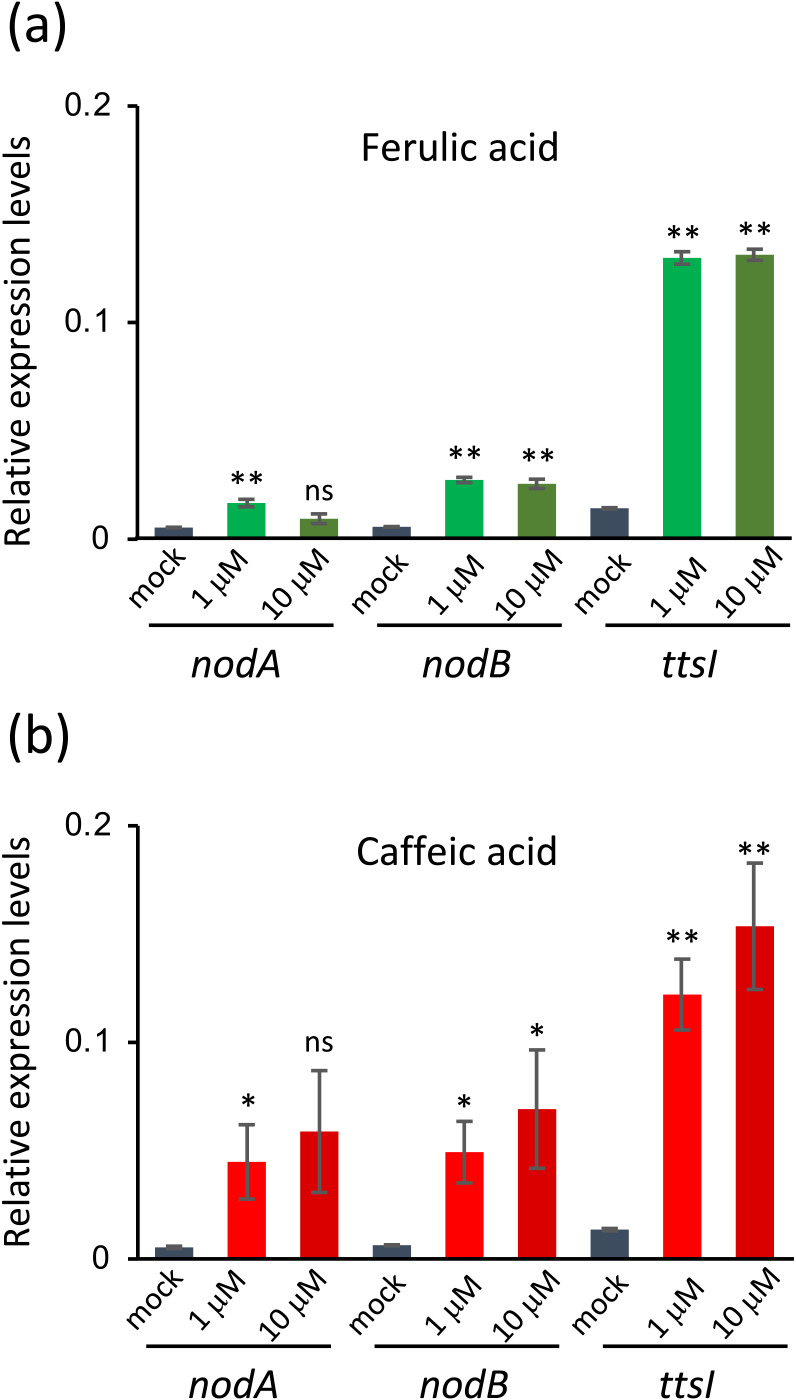
qRT-PCR ana­lysis of the expression of *nodA*, *nodB*, and *ttsI* genes in *Mesorhizobium japonicum* MAFF303099 harboring p*Mj*-NodD1 in the absence or presence of (a) ferulic acid or (b) caffeic acid. Error bars show S.E. (*n*=3 or 4). Significant differences between the absence (mock) and presence of phenolic acids were assessed by the Student’s *t*-test (** *P*<0.01, * *P*<0.05, *ns*, not significant).

We then conducted qRT-PCR to investigate whether phenolic acids induce the transcriptional activation of the NF biosynthesis genes *nodA* and *nodB* using the ML033/pMj-NodD1 strain. The *nodA* gene was significantly induced by 1‍ ‍μM ferulic acid (**6**) and caffeic acid (**5**), while the *nodB* gene was significantly induced at both 1 and 10‍ ‍μM, similar to the *ttsI* gene (Fig. 5). Caffeic acid (**5**) exerted similar effects to ferulic acid (**6**) (Fig. 5). As in the β-galactosidase assay, cinnamic acid (**3**) did not induce *nodA*, *nodB*, or *ttsI* expression (data not shown). The above results revealed that the *M. japonicum* NodD1 receptor recognizes phenolic acids, such as ferulic and caffeic acids, and activates the transcription of the *nod* genes.

### Phenolic acids exuded from legume roots

To elucidate whether phenolic compounds were exuded from the roots of *L. japonicus*, we used the SRM mode of UPLC-TQMS to analyze the components of culture media 7 days after hydroponic cultures of *L. japonicus*, red clover, and alfalfa. The levels of five phenolic acids that induce the production of NFs (*p*-coumaric acid [**4**], caffeic acid [**5**], ferulic acid [**6**], 5-hydroxyferulic acid [**7**], and phloretic acid [**13**]) and those of phenolic acids that do not (cinnamic acid [**3**] and sinapic acid [**8**]) were quantified. *p*-Coumaric acid (**4**) and ferulic acid (**6**) were detected in hydroponic media from all three plant species, with the levels of *p*-coumaric acid (**4**) being higher in exudates from *L. japonicus* (Table 2). Cinnamic acid (**3**) was only secreted by *L. japonicus* (Table 2). Trace levels (0.2‍ ‍nmol g^–1^ FW plants or 0.6‍ ‍nmol mg^–1^ root exudate) of caffeic acid (**5**), sinapic acid (**8**), and phloretic acid (**13**) were detected, whereas 5-hydroxyferulic acid (**7**) was undetectable (less than 0.2‍ ‍nmol g^–1^ FW plant or 0.6‍ ‍nmol mg^–1^ root exudate) in either legume.

To monitor the induction of the *ttsI*::*lacZ* fusion in ML033 series strains in the rhizosphere of *L. japonicus*, approximately 10^8^ rhizobial cells were spread on the roots of 10-day-old seedlings grown on agar medium with X-Gal. The inoculation with the ML033/p*Mj*-NodD1 strain resulted in X-gal blue staining on and around the root surface (Supplementary Fig. S3). The inoculation of the ML033/p*Mj-*NodD2 strain resulted in X-gal staining only on the root surface. These results strongly suggest that phenolic acid(s) activating NodD1 were exuded not only on the root surface, but also into the rhizosphere of *L. japonicus*.

## Discussion

The *nod* gene inducer in *L. japonicus*–*Mesorhizobium* symbiosis has not been identified despite decades of research. Therefore, we developed a highly sensitive method to analyze LCOs using UPLC-TQMS. MS was previously applied to the study of LCOs, mainly for a structural ana­lysis (Niwa *et al.*, 2001; Bek *et al.*, 2010). To use MS for screening, we simplified purification and reduced the required culture volume, which allowed us to evaluate 40 phenolic compounds. Among them, we identified five phenolic acids that had the potential to induce LCO production in *M. japonicum* MAFF303099. The production of LCOs was enhanced by increases in the concentrations of each of these phenolic acids. LCOs produced by *M. japonicum* MAFF303099 in the presence of caffeic acid (**5**) induced root hair deformation, and nodule numbers in *L. japonicus* inoculated with *M. japonicum* were increased by the addition of ferulic acid (**6**) and caffeic acid (**5**). These results clearly identified phenolic compounds, but not flavonoids, as *nod* gene inducers. Phenolic acids are produced via shikimic acid through the phenylpropanoid pathway, and also as intermediates of the monolignol pathway in vascular plants. A previous study reported that rhizobia utilized phenolic acids as carbon sources (Blum *et al.*, 2000). A number of *nod* gene inducers have been identified in legumes, and most of them are (iso)flavonoids (Liu and Murray, 2016); to the best of our knowledge, this is the first study to demonstrate that phenolic acids function as *nod* gene inducers.

**Fig. 6.**
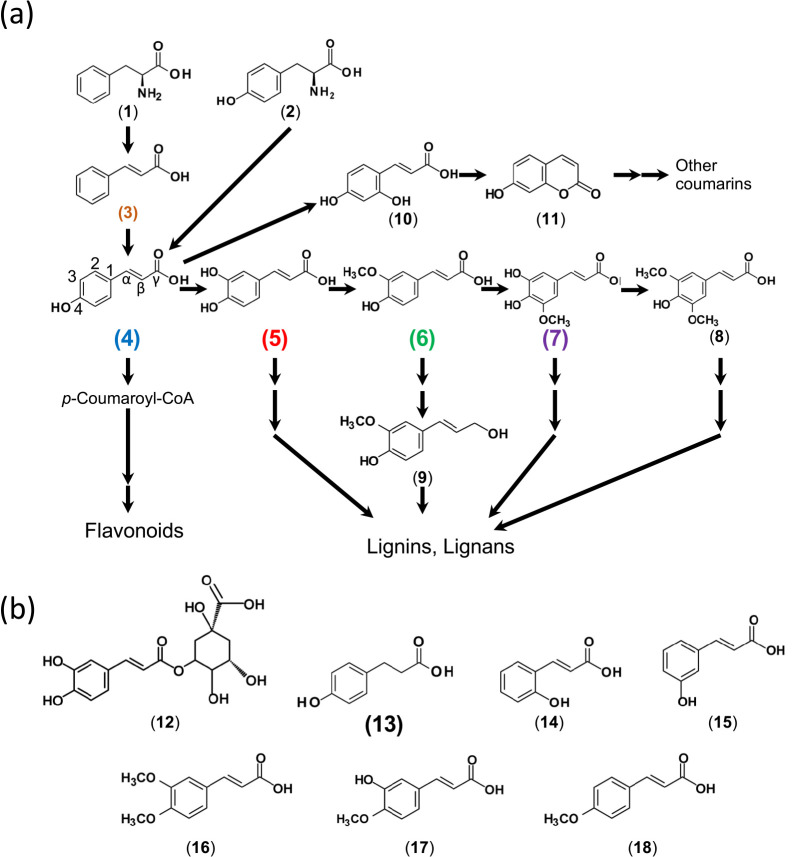
Chemical structures of phenolic acids used in the present study. (a) Phenolic acids in the major phenylpropanoid pathway towards coumarins, lignins, and lignans. (b) Other phenolic acids. See Table 1 for compound names.

The identified candidate *nod* gene inducers of *L. japonicus* are phenylpropanoids with a carboxylic acid group, in contrast to coniferyl alcohol (**9**) and chlorogenic acid (**12**) (Fig. 6). A comparison with phenolic acids that did not induce LCOs (Table 2), *i.e.* l-phenylalanine (**1**), *trans*-cinnamic acid (**3**), umbellic acid (**10**), *o*-coumaric acid (**14**), *m*-coumaric acid (**15**), 3,4-dimethoxycinnamic acid (**16**), isoferulic acid (**17**), and *p*-methoxycinnamic acid (**18**), suggested that a hydroxyl at C-4 and a hydrogen at C-2 or C-6 are important for the ability to induce NFs in *M. japonicum* (Fig. 6). A single methoxy group did not preclude LCO-inducing activity, whereas two methoxy groups, as in sinapic acid (**8**), did. A double bond between α and β carbons appears to be important for the induction of LCO production, but is not essential because weak induction was detected with phloretic acid (**13**). Therefore, the basic structure of a *nod* gene inducer appears to be that of *p*-coumaric acid (**4**) with at most a single methoxy group at C-3 or C-5 and hydrogens at C-2 and C-6. We suggest that the carboxylic acid group and the C-3, C-4, and C-5 positions of the phenyl ring were recognized by NodD.

Aldonic acids have been shown to promote LCO biosynthesis in *Mesorhizobium* strains (Gagnon and Ibrahim, 1998). In our experiments, none of the three aldonic acid compounds tested induced LCO production at the concentrations at which the five phenolic acids induced it. Gagnon and Ibrahim (1998) identified aldonic acids in the root exudates of *Lupinus albus* by screening based on measurements of the β-galactosidase activities of *Rhizobium lupini* strains harboring *nodC*::*lacZ* fusions, and 10‍ ‍mM tetronic acid was required to induce detectable LCO production in *M. japonicum* R7A. We only tested concentrations up to 100‍ ‍μM, which may explain why the aldonic acids tested did not induce LCOs. Since tetronic acid was not detected in the root exudates or seed metabolites of *L. japonicus* (Hashiguchi *et al.*, 2018), aldonic acids cannot be endogenous *nod* gene inducers of *L. japonicus*.

In contrast to aldonic acids, the presence of *p*-coumaric acid (**4**), ferulic acid (**6**), and *trans*-cinnamic acid (**3**) was confirmed in the root exudates of *L japonicus* (Table 2). In addition, *p*-coumaric acid (**4**) and ferulic acid (**6**) are listed as metabolites in the seeds of experimental and wild accessions of *L. japonicus* in LegumeBase, the resource database of National BioResource Project *Lotus*/*Glycine* (Hashiguchi *et al.*, 2018). Phenolic acids have also been reported in the root exudates of other legume and non-legume plants (Mandal *et al.*, 2010). We identified *p*-coumaric acid (**4**) and ferulic acid (**6**) in the root exudates of *T. pratense* and *M. sativa* (Table 2), and, thus, these phenolic acids do not appear to contribute to host specificity. Since phenolic acids are generally present in the rhizosphere, the responses of *Mesorhizobium* strains to them may contribute to their associations with a broad range of plants, including non-host plants. *M. japonicum* MAFF303099 associates with non-host plants, such as *Arabidopsis thaliana* (Poitout *et al.*, 2017), as a root epiphyte. T3SS may play a role in this relationship, as reported in *Bradyrhizobium* strains (Piromyou *et al.*, 2015). In the present study, the expression of *ttsI*, a regulator of the T3SS gene cluster, was induced by phenolic acids at higher levels than that of *nodA* in *M. japonicum* MAFF303099 carrying p*Mj*-NodD1 (Fig. 5). Therefore, phenolic acid recognition by *Mesorhizobium* strains may have a function against non-host plants by inducing T3SS. Regarding host specificity, differences in the concentrations of phenolic acids in root exudates may affect host specificity in *L. japonicus*–*Mesorhizobium* symbiosis because the concentration of *p*-coumaric acid (**4**) in the root exudates of *L. japonicus* was more than ten-fold higher than those in the root exudates of red clover and alfalfa (Table 2). The NF receptors of host plants are important for recognition that affects the host range in plant–rhizobia symbiotic interactions (Radutoiu *et al.*, 2007; Bek *et al.*, 2010). We confirmed the function of LCOs induced by caffeic acid (**5**) as endogenous NFs for *L. japonicus* by demonstrating their ability to induce root hair deformation (Supplementary Fig. S2). The number of mature nodules was increased by the addition of phenolic acid-type *nod* gene inducers together with an inoculation with *M. japonicum* (Fig. 3), as previously reported in pea and soybean (Novák *et al.*, 2002; Pan *et al.*, 2008). Therefore, the recognition of phenolic acids by *Mesorhizobium* strains may function in two ways: the production of T3SS may contribute to associations with a wide range of plants, and, at the same time, the production of LCOs may function in host recognition in symbiotic interactions.

Although we confirmed LCO production after treatments with five phenolic acids, we failed to detect the induction of *nod* genes by RT-PCR or *ttsI* expression using its promoter fused to *lacZ* in the genome (Fig. 4a). The transcript levels of genes regulated by NodD may be below the detection level of normal RT-PCR and a single copy of *lacZ* in the genome of *M. japonicum* MAFF303099; this may explain our failure to identify *nod* gene inducers in *L. japonicus–Mesorhizobium* symbiosis, even though it has been widely used as a model of symbiosis in legume plants (Liu and Murray, 2016). The *nodA* promoter fused to *lacZ* in a multicopy plasmid (pMP220) has been used to detect *lacZ* expression in *M. japonicum* MAFF303099 (Kojima *et al.*, 2012). We adopted this approach and attempted to increase the copy number of *nodD* genes by introducing *nodD1* or *nodD2* into a multicopy plasmid. This ana­lysis revealed that NodD1 was more sensitive to phenolic acids than NodD2, indicating a functional differentiation between NodD1 and NodD2 with regards to the perception of phenolic acid signals. In *M. japonicum* R7A, NodD1 and NodD2 are functionally redundant for nodulation, with *nodD1* mutants exhibiting only a slight delay in nodulation (Rodpothong *et al.*, 2009). Kelly *et al.* (2018) showed the preferential activation of NodD1 and NodD2 by different compounds produced at defined stages of symbiotic infection. NodD1 is primarily involved in the induction of downstream genes within root hair infection threads. Since phenolic acids are intermediates in the biosynthesis of a number of phenolic compounds, such as flavonoids and monolignols, it is reasonable that they act as *nod* gene inducers in root hair infection threads. However, we detected *p*-coumaric acid (**4**) and ferulic acid (**6**) in the root exudates of *L. japonicus* (Table 2) and *lacZ* gene induction in the rhizosphere using an *M. japonicum* strain with the *nodD1* plasmid (Supplementary Fig. S3). Therefore, the *nod* gene–inducing activities of phenolic acids are not restricted to root hair infection threads, they may also be involved in a wide range of associations in the rhizosphere.

In the present study, we used the direct detection of LCOs to screen for *nod* gene inducers in *L. japonicus*–*Mesorhizobium* symbiosis. We identified five candidate compounds in the group of phenolic acids, and detected two in the root exudates of *L. japonicus*. By increasing the copy number of one of the two *nodD* genes in *M. japonicum*, we revealed that phenolic acids as *nod* gene inducers were mainly recognized by NodD1. Overall, we propose that phenolic acids are a novel type of *nod* gene inducer in the *L. japonicus*–*Mesorhizobium* symbiosis system. Therefore, substances that act as mutual symbiotic signals from both sides, *L. japonicus* and *M. japonicum*, are now revealed. The present results will accelerate the elucidation of the regulatory mechanisms in this symbiotic system.

## Citation

Shimamura, M., Kumaki, T., Hashimoto, S., Saeki, K., Ayabe, S., Higashitani, A., et al. (2022) Phenolic Acids Induce Nod Factor Production in *Lotus japonicus*–*Mesorhizobium* Symbiosis. *Microbes Environ ***37**: ME21094.

https://doi.org/10.1264/jsme2.ME21094

## Supplementary Material

Supplementary Material

## Figures and Tables

**Table 1.** NF (LCO)-inducing activities of compounds tested in the present study.

Class	Compound	PubChem CID	Activity*
Phenylpropanoids	l-Phenylalanine (**1**)	6140	–
	l-Tyrosine (**2**)	6057	–
	*trans*-Cinnamic acid (**3**)	444539	–
	*p*-Coumaric acid (**4**)	637542	+
	Caffeic acid (**5**)	689043	+
	Ferulic acid (**6**)	445858	++
	5-Hydroxyferulic acid (**7**)	446834	+
	Sinapic acid (**8**)	637775	–
	Coniferyl alcohol (**9**)	1549095	–
	Umbellic acid (**10**)	446611	–
	Umbelliferone (**11**)	5281426	–
	Chlorogenic acid (**12**)	1794427	–
	Phloretic acid (**13**)	10394	+
	*o*-Coumaric acid (**14**)	637540	–
	*m*-Coumaric acid (**15**)	637541	–
	3,4-Dimethoxycinnamic acid (**16**)	717531	–
	Isoferulic acid (**17**)	736186	–
	*p*-Methoxycinnamic acid (**18**)	699414	–
Chalcones	Butein	5281222	–
	Isoliquiritigenin	638278	–
Flavanones	(*2S*)-Eriodictyol	440735	–
	(*2S*)-Naringenin	439246	–
Flavones	Apigenin	5280443	–
	Luteolin	5280445	–
Flavonols	Gossypetin	5280647	–
	Herbacetin	5280544	–
	Isorhamnetin	5281654	–
	Kaempferol	5280863	–
	Myricetin	5281672	–
	Quercetin	5280343	–
Isoflavones	Biochanin A	5280373	–
	Daidzein	5281708	–
	Formononetin	5280378	–
	Genistein	5280961	–
	Pseudobaptigenin	5281805	–
Isoflavans	(*3R*)-Vestitol	182259	–
Coumestans	Coumestrol	5281707	–
Aldonic acids	Erythronic acid (lactonized)	5325915	–
	Succinic anhydride	7922	–
	Tetronic acid (lactonized)	521261	–

*: ++ and + indicate LCO-inducing activity at 0.3‍ ‍mg L^–1^ (++) and 15‍ ‍mg L^–1^ (+); – no activity.

**Table 2.** Characteristics of hydroponic cultures and phenolic acid contents in root exudates of three leguminous plants. Values are mean total contents±S.E. (*n*=4).

	Seed germinated (g DW)	Plant FW (g)	Root exudates (mg)	Contents (nmol g^–1^ FW plant)				Contents (nmol mg^–1^ root exudate)		
				**3**	**4**	**6**		**3**	**4**	**6**
*Lotus japonicus*	0.82	1.44	2.7	4.0±0.1	11.9±0.3	2.0±0.0		21.1±0.6	63.2±1.3	10.5±0.1
red clover	0.69	3.38	2.7	n.d.	0.3±0.0	1.7±0.0		n.d.	4.0±0.0	21.6±0.1
alfalfa	2.07	10.21	8.2	n.d.	0.4±0.0	0.6±0.0		n.d.	5.0±0.0	8.0±0.1

FW, fresh weight. **3**, *trans*-cinnamic acid; **4**, *p*-coumaric acid; **6**, ferulic acid; n.d., not detected.
