# Supplementary material for: Phenolic Acids Induce Nod Factor Production in Lotus japonicus–Mesorhizobium Symbiosis
Source: Microbes Environ. 2022 Mar 12;37(1):ME21094. doi: 10.1264/jsme2.ME21094 (PMC8958295; doi:10.1264/jsme2.ME21094)
Supplement: Supplementary file 1 — Supplementary Material [file 37_21094_s1.pdf]

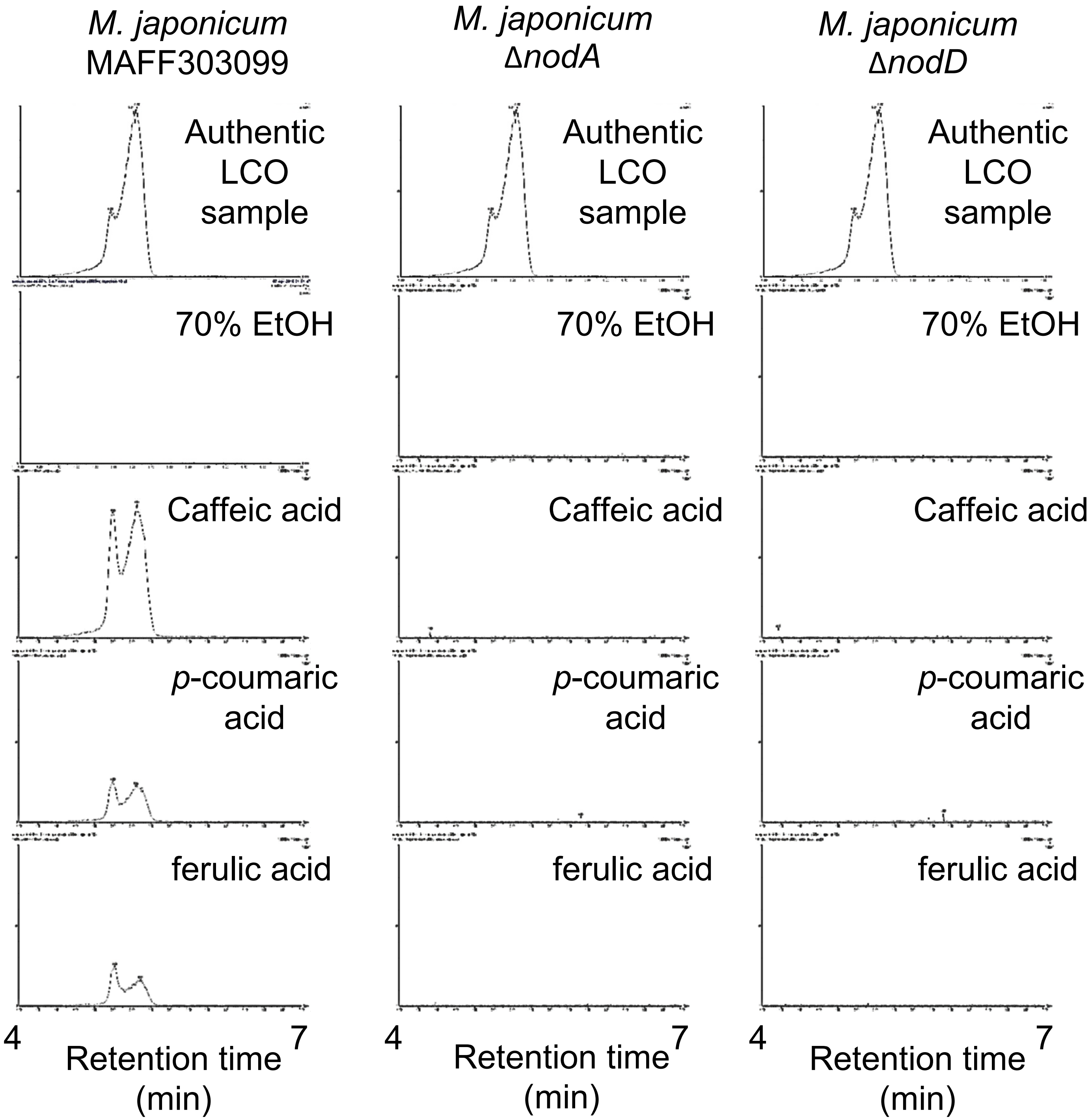

Supplementary figure S1. LCO production using *M. japonicum* MAFF303099, and *nodA* and *nodD* deletion variants. *M. japonicum* MAFF303099 series were cultured in a TY medium supplemented with mock, 100 μM caffeic acid, *p*-coumaric acid or ferulic acid for 66 h, and the LCO productivity was analyzed by SRM method of UPLC-MS/MS as shown in Figure 1.

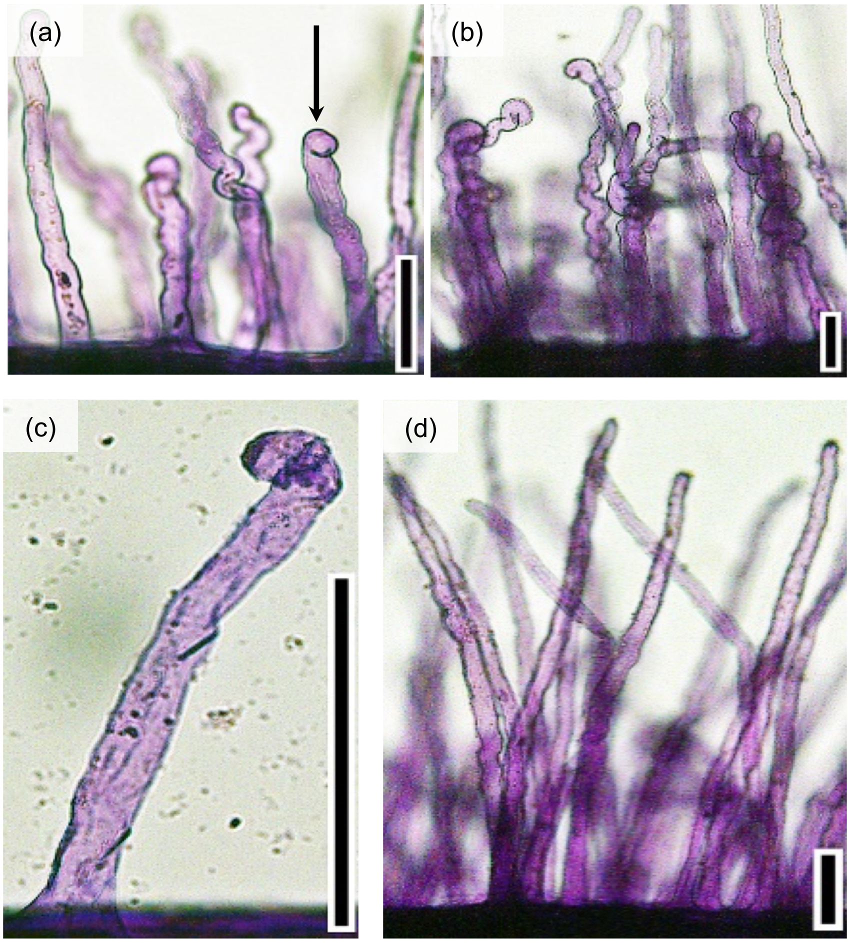

Supplementary figure S2. Root hair deformation of *L. japonicus* B-129 Gifu caused by caffeic acid induced LCOs of *M. japonicum* MAFF303099. Four-day-old dark-grown plants were treated with 10^{-9} M LCOs (a, b, c) or mock (d) for 24 h. The arrow and (c) indicate the curling root hair tips. Scale bars = 50 (a, d), 30 (b) and 10 (c) μm.

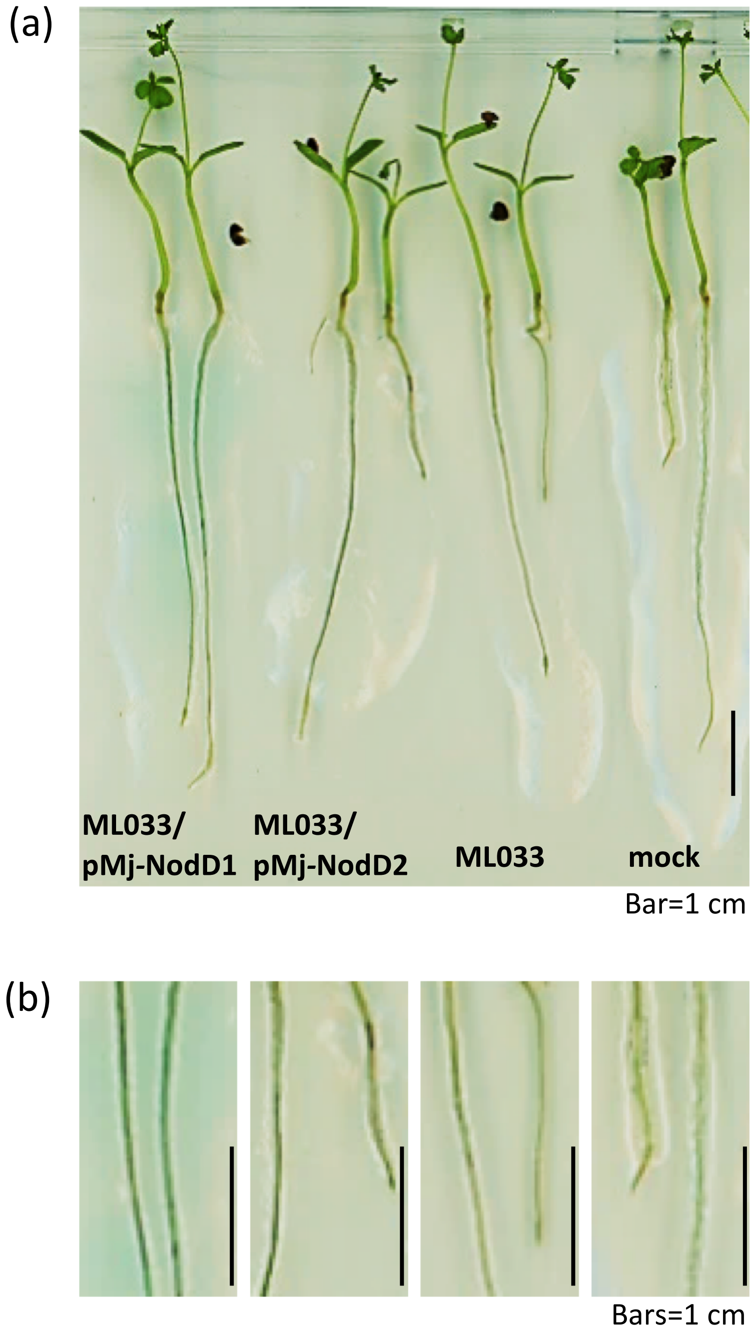

Supplementary figure S3. X-GAL staining of infection with *M. japonicum* ML033, ML033 / p*Mj*-NodD1 and ML033 / p*Mj*-NodD2 on *Lotus japonicus* MG-20 seedlings grown on agar plates. (a) X-gal blue staining was observed on and around the root surface spread with ML033 / p*Mj*-NodD1, and on only root surface with ML033 / p*Mj*-NodD2. (b) Superimposed of root surface in panel (a).

Supplementary Table S1. Bacterial stains and plasmids.

Strain or plasmid	Characteristic*	Source or reference
**Strains**		
Mesorhizobium japonicum		
MAFF303099	Wild type strain, Pm^r	Saeki and Kouchi 2000, Kaneko *et al.*, 2000
MAFF303099-pMP2112	MAFF303099 carrying pMP2112, Pm^r, Sp^r	This study
MAFF303099 Δ*nodA*	MAFF303099 derivative, *NodA::*Spectinomycin resistance gene, Pm^r, Sp^r	This study
MAFF303099 Δ*nodD*	MAFF303099 derivative, *NodD1*(mll6179)-*nolL* (mlr8757)-*nodD2* (mlr6182)-mll6183-mlr6185::Kanamycin resistance gene,Pm^r, Km^r	This study
ML033	MAFF303099 derivative, fusion fragment of *ttsI* (*mlr6334*) promoter and *lacZ* was integlated into MAFF303099 chromosome, Pm^r, Tc^r	Okazaki *et al.*, 2010
ML033-pMj-NodD1	ML033 carrying pMj-NodD1, Pm^r, Tc^r, Km^r	This study
ML033-pMj-NodD2	ML033 carrying pMj-NodD2, Pm^r, Tc^r, Km^r	This study
Mesorhizobium loti		
TONO	Wild type strain	Kawaguchi *et al.*, 2002, Shimoda *et al.*, 2016
***Mesorhizobium* spp.**		
Bishamon 1-c2	Isolate from nodule on *Lotus japonicus* grown up in the wild (Bishamon, 34° 54'10.3"N 139°53'15.7"E)	This study
Nojimazaki 1-a1	Isolate from nodule on *L. japonicus* grown up in the wild (Nojimazaki, 35° 08'26.5"N 139°39'36.0"E)	This study
Escherichia coli		
DH5α	Cloning host	Takara Bio
**Plasmids**		
Cosmid c243	Cosmid carrying MAFF303099 chromosomal fragment containing the *nodD1*(mll6179)-*nolL* (mlr8757)-*nodD2* (mlr6182)-mll6183-mlr6185 region, Tc^r	Hattori *et al.,* 2002
Cosmid c242.1	Cosmid carrying MAFF303099 chromosomal fragment containing the *nodA*(mlr8755), Tc^r	Hattori *et al.,* 2002
pUCKM1	Source of kanamycin resistance gene, Km^r, Ap^r	Saeki *et al.,* 1991
pEMA49	Cosmid c243 derivative, *nodD*1(mll6179)-*nolL* (mlr8757)-*nodD2* (mlr6182)-mll6183-mlr6185 region was replaced to kanamycin resistance gene, Tc^r, Km^r	This study
pKST001R	pCR2.1 containing *aadA* flanked by FRTs, Ap^r, Km^r, Sp^r	Hanyu *et al.,* 2009
pML8755DA	Cosmid c242.1 derivative, *NodA* was replaced to spectinomycin resistance gene, Tc^r, Sp^r	This study
pMP2112	pMP2733 containing the *Rhizobium leguminosarum* bv. trifolii *nodD*, Sp^r	Lopez-Lara *et al.*, 1995
pBBR1MCS-2	Cloning vector, Km^r	Kovach *et al.*, 1995
pMj-NodD1	pBBR1MCS-2 carrying 1272-bp fragment containing the coding region and promoter region of MAFF303099 *nodD1* (*mlr6182*), Km^r	This study
pMj-NodD2	pBBR1MCS-2 carrying 1365-bp fragment containing the coding region and promoter region of MAFF303099 *nodD2* (*mll6179*), Km^r	This study

*Pm^r, Sp^r, Tc^r, Km^r and Ap^r indicate resistance to phosphomycin, spectinomycin, tetracycline, kanamycin and ampicillin, respectively.

Supplementary Table S2. Primers used in the present study.

Oligonucleotides	Sequence
nodA(mlr8755)_F	5'-TGGATGCTCGCGATCTAAAC-3'
nodA(mlr8755)_R	5'-TTTTTCGGGTTCTTTTGTGC-3'
nodB(mlr6175)_F	5'-ATACTCGATGTGCTGGCGCAAAAT-3'
nodB(mlr6175)_R	5'-GCCTGGTTCGCCTCAAATACTTCAC-3'
ttsl(mlr6334)_F	5'-ATTGGCTGAAGCAGCTAAGG-3'
ttsl(mlr6334)_R	5'-GGCTTGAGCAGAAAATCGTC-3'
16S(rrn16Sa)_F	5'-AGCATTCAGTTGGGCACTCT-3'
16S(rrn16Sa)_R	5'-TCCGAACTGAGATGGCTTTT-3'
rpoA_F	5'-TGAACATCAAGGAGATCGCCA-3'
rpoA_R	5'-AGCGTGCAGATGACGTGGTC-3'
wan_mlr8755_upper	5'-AGATCAGGCTTTGAAAGGCATGGTTTTAACATCCATAGCGTGGATATTCCGGGGATCCGTCGACC-3'
wan_mlr8755_lower	5'-CGCGCTGACTGACGTCGTAGCGGGCGTCCTTCATAGCTCTGGACCTGTAGGCTGGAGCTGCTTCG-3'
KS_nodSJ_F01S	5'-CGCGTCGATCAATGAAGACTGC -3'
KSnodC_Rev01	5'-GTAGATTCGGACACGATACTCGC -3'

Supplementary Table S3. SRM conditions of UPLC-TQMS.

	Retention time (min)	molar mass	ESI (m/z)<br>(Cone voltage)	ESI (m/z)<br>(Collision energy)	mode
Caffeic acid	4.01	180.16	181<br>(20)	163<br>(10)	+
-hydroxyferulic ac	4.51	210.05	211<br>(20)	193<br>(10)	+
Phloretic acid	5.48	166.17	165<br>(20)	121<br>(10)	-
p -Coumaric acid	5.80	164.16	165<br>(20)	147<br>(10)	+
Ferulic acid	7.02	194.18	195<br>(20)	177<br>(10)	+
Sinapic acid	7.31	224.21	225<br>(20)	207<br>(10)	+
Cinnamic acid	8.90	148.16	149<br>(20)	103<br>(20)	+
